# Case Report: A structured point-of-care ultrasound pathway for the crashing neonate in the NICU: a case series and algorithm proposal

**DOI:** 10.3389/fped.2026.1787858

**Published:** 2026-06-04

**Authors:** Bang Du, Min-Ling Mai, Jin-Feng Li, Hao-Qiang Xie, Jin-Gen Lie, Bi-Ying Deng, Xiao-Guang He

**Affiliations:** Department of Neonatology, Dongguan Children’s Hospital Affiliated to Guangdong Medical University, Shilong Town/Dongguan, Guangdong, China

**Keywords:** bedside diagnosis, intraventricular hemorrhage, neonatal intensive care, pericardial tamponade, point-of-care ultrasound, tension pneumothorax

## Abstract

Point-of-care ultrasound (POCUS) is increasingly recognized as an essential diagnostic tool in the neonatal intensive care unit (NICU), often described as the “stethoscope of the modern era”. It provides real-time, bedside imaging that enhances clinical assessment and guides urgent intervention. Sudden clinical deterioration in neonates—commonly due to tension pneumothorax, pericardial tamponade, massive pleural effusion, or severe intracranial hemorrhage—requires prompt diagnosis and management. This retrospective case series illustrates the utility of POCUS in three critically ill neonates: one with postoperative tension pneumothorax, another with umbilical venous catheter-associated pericardial tamponade, and a third with severe intraventricular hemorrhage in an extremely preterm infant. In each case, POCUS enabled rapid diagnosis, guided immediate intervention, and influenced outcomes. We also review the literature supporting POCUS integration into NICU practice and propose a structured screening protocol for neonates with acute cardiopulmonary decompensation. This series underscores the role of POCUS as a first-line imaging tool in neonatal emergencies and underscores the imperative for standardized training to ensure its effective and responsible use (diagnostic stewardship) within the NICU.

## Introduction

Point-of-care ultrasound (POCUS) is a dynamic imaging tool that immediately provides diagnostic information, complementing traditional physical exams and history-taking (1). In the neonatal intensive care unit (NICU), where clinical status can change rapidly, POCUS offers a valuable tool for identifying life-threatening conditions and guiding time-sensitive interventions (2, 3).

Neonates are particularly vulnerable to acute cardiopulmonary decompensation due to physiological immaturity and the frequent use of invasive supports such as mechanical ventilation and central venous catheters. Common causes include tension pneumothorax (4, 5), pericardial effusion with tamponade (6, 7), large pleural effusions, and severe intracranial hemorrhage. Timely differentiation among these conditions is critical, as each demands a distinct management approach.

POCUS has become a feasible and accurate bedside tool in neonatology. Growing evidence supports its role in improving diagnostic accuracy, reducing radiation exposure from radiography, and enhancing clinician confidence. Notably, multicenter studies have firmly validated the reliability of lung ultrasound in diagnosing pneumothorax among critically ill neonates (8).

While comprehensive POCUS algorithms like SAFE-R (9, 10) have been developed to cover a broad spectrum of etiologies, their abdominal and aortic screening steps may require probe pressure on the fragile abdominal wall, which could be of particular concern in extremely low birth weight infants. The NICU population includes a substantial proportion of extremely low birth weight (ELBW) infants, who are uniquely vulnerable to procedural stress and handling-induced intracranial hemorrhage. Therefore, a screening protocol for the crashing neonate must balance diagnostic breadth against iatrogenic risks. We propose a streamlined, “lightweight” three-step “heart-lungs-brain” pathway. This algorithm specifically targets the three most time-critical causes of sudden deterioration—pneumothorax, pericardial tamponade, and severe intraventricular hemorrhage (IVH)—minimizing stress while expediting life-saving interventions.

This case series presents three neonates who experienced sudden deterioration in the NICU, in which this streamlined POCUS approach played a critical diagnostic role. We also review relevant literature to support the implementation of this practical screening algorithm for unstable neonates.

## Case presentation

This retrospective case series included three neonates in our hospital's NICU who experienced sudden clinical deterioration and underwent POCUS evaluation between January 2020 and December 2023. The Ethics Committee of Dongguan Children's Hospital approved the study (Approval No.: LL2022112901). Informed consent was obtained from the parents or guardians. Attending neonatologists performed all POCUS examinations using a standardized PHILIPS CX-50 system equipped with a high-frequency linear transducer (9–13 MHz). All POCUS examinations were performed by neonatologists who completed standardized training (theory, hands-on, and exam) and received certification from the Chinese Critical Ultrasound Study Group. Our department is a lung ultrasound training base in South China, and all neonatologists are competent in lung ultrasound.

Based on established rapid screening frameworks for suddenly decompensating infants, such as the SAFE-R (Sonographic Assessment of life-threatening Emergencies - Revised) protocol (9), our POCUS protocol was structured to quickly rule in or rule out life-threatening conditions. The systematic scanning involved: (1) Cardiac ultrasound using standard apical and subcostal views to rapidly assess cardiac contractility and rule out pericardial tamponade; (2) Lung ultrasound scanning the anterior, lateral, and posterior chest regions bilaterally to evaluate for tension pneumothorax and massive pleural effusion; and (3) Cranial ultrasound performed via the anterior fontanelle to screen for severe intraventricular hemorrhage. Of note, our proposed algorithm explicitly excludes routine abdominal and aortic screenings from the primary emergency sequence. This modification is designed to limit prolonged manipulation and excessive probe pressure, thereby mitigating the risk of exacerbating intracranial hemorrhage in extremely preterm infants. Abdominal POCUS is reserved as a secondary step only if the initial cardiopulmonary and cranial assessments are unyielding, or if specific abdominal signs are clinically evident. Ultimately, all POCUS findings were interpreted in conjunction with the neonate's clinical manifestations.

### Case 1

The infant was a 34-week preterm neonate transferred to our NICU after congenital esophageal atresia surgery. The infant was diagnosed with congenital esophageal atresia with tracheoesophageal fistula (type IIIB) and underwent end-to-end esophageal anastomosis and repair of the tracheoesophageal fistula on the 5th day after birth. Mechanical ventilation was used in the early postoperative period, and was switched to high-flow nasal cannula (HFNC) on the 5th day after surgery. The chest tube was removed on the 18th postoperative day. After removal of the chest tube, lung ultrasound examination revealed lung sliding sign and B-lines.

**Figure 1:**
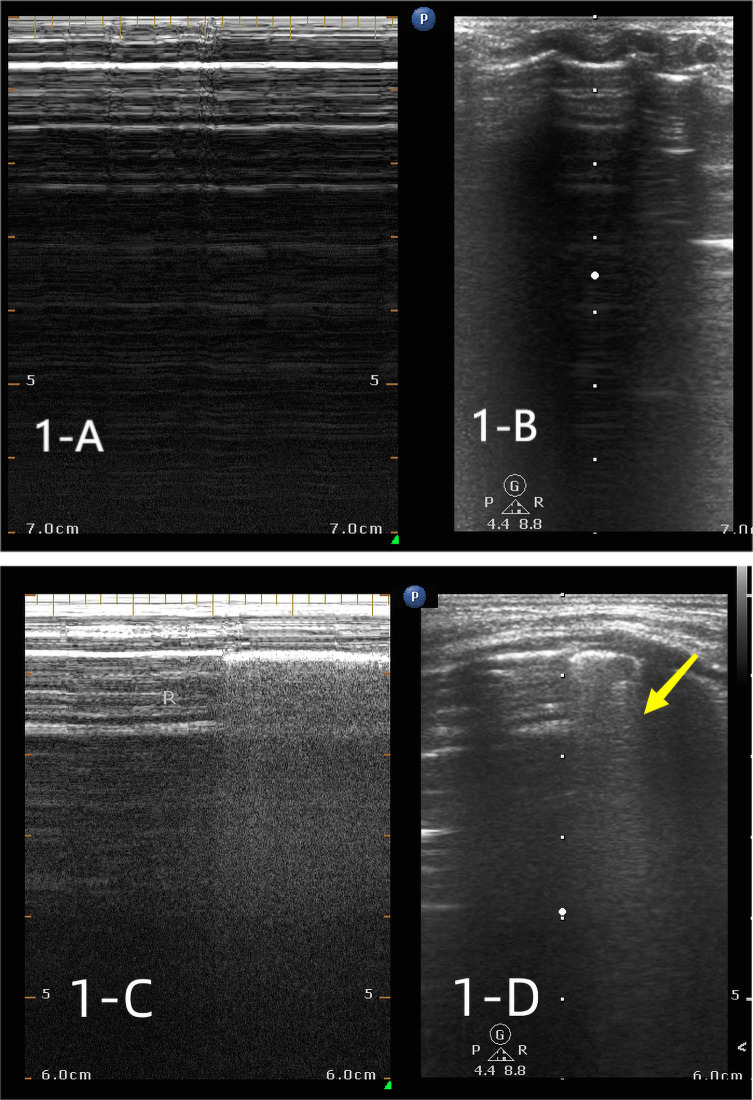
Lung ultrasound in neonatal pneumothorax. **(A)** M-mode showing stratospheric sign. **(B)** B-mode image representing the anterior chest wall, where absent lung sliding was dynamically observed in real-time during the examination. **(C,D)** Post-drainage reappearance of lung sliding and B-lines (yellow arrows).

Two hours after thoracic drain removal, the infant developed respiratory distress, with transcutaneous oxygen saturation (TcSO₂) at 70%, diminished breath sounds in both lungs (more pronounced on the right), heart rate of 80 beats per minute, and distant heart sounds on auscultation. Lung POCUS showed loss of heart and thymus shadows, absence of lung sliding and B-lines on the right side, and no lung point. M-mode ultrasound demonstrated the “stratosphere sign,” suggesting a large pneumothorax (Figures 1A,B).

Immediate endotracheal intubation with positive-pressure ventilation and cardiopulmonary resuscitation were initiated, along with emergency needle thoracentesis, during which 40 mL of air was aspirated, followed by reinsertion of a chest tube. Figures 1A,B demonstrated the classic signs of pneumothorax, which directly led to the decision for immediate needle thoracentesis. After treatment, the infant's heart rate increased to 108 beats per minute, and TcSO₂ improved to 96%. Follow-up lung ultrasound revealed a lung point at the right midclavicular line, indicating reduction of the pneumothorax (Figures 1C,D).

### Case 2

**Figure 2:**
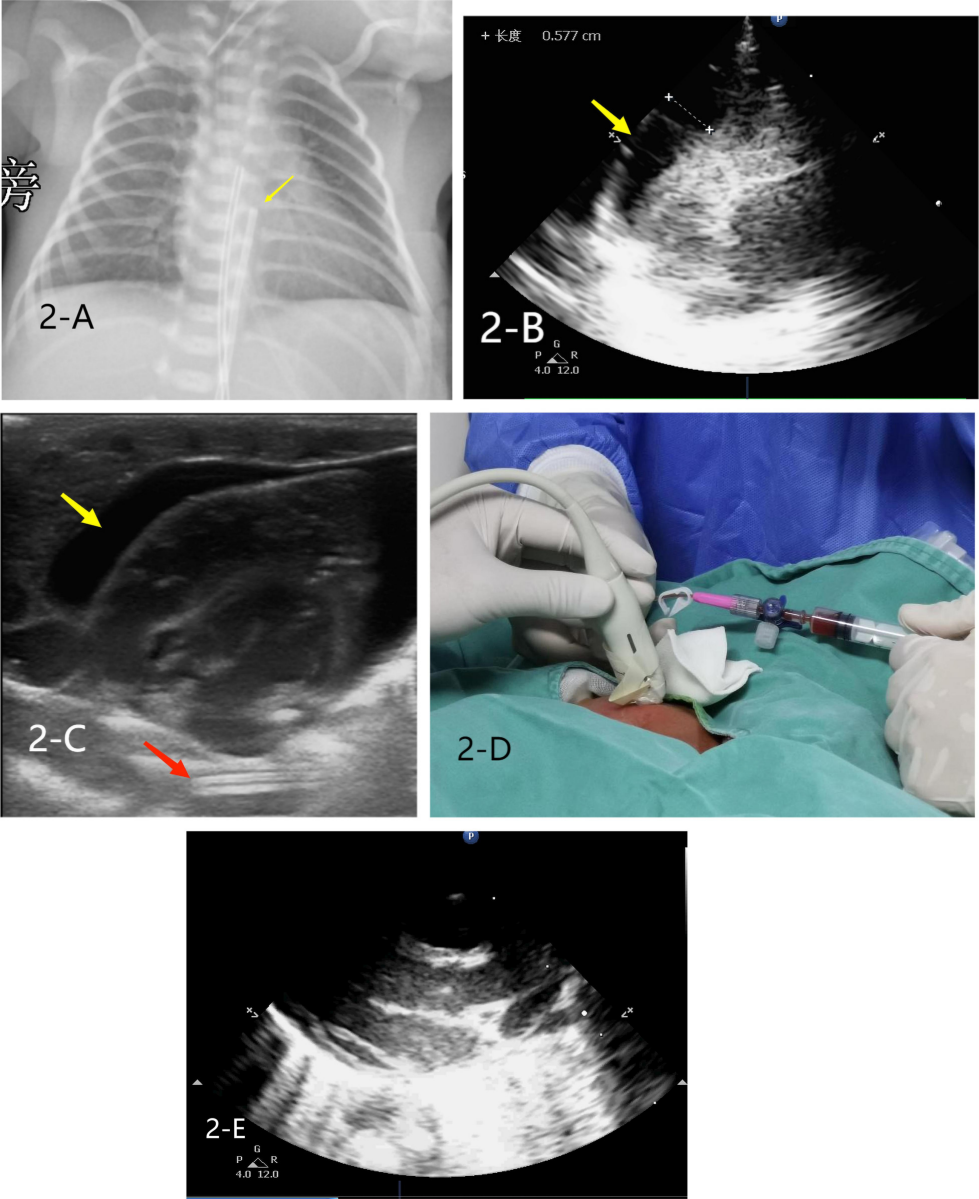
Cardiac POCUS in pericardial tamponade. **(A)** The tip of the umbilical vein is located at the superior border of T7. **(B,C)** Apical view showing pericardial effusion (yellow arrow), with the umbilical arterial catheter tip clearly visible in the descending aorta (red arrow) and the UVC tip poorly visualized. **(D)** Ultrasound-guided pericardiocentesis. **(E)** Post-drainage resolution of effusion.

The patient was a female infant born extremely preterm at 25 + 5 weeks’ gestation, weighing 900 g, with no history of perinatal asphyxia. After delivery, the infant was placed on mechanical ventilation. At 6 h after birth, an umbilical venous catheter (UVC) was inserted. The insertion depth was calculated using the Shukla formula (3 ×  birth weight in kg + 9)/2 + 1 plus the length of the umbilical stump, resulting in an initial depth of 8 cm. The immediate post-procedure chest x-ray confirmed that the catheter tip was located at the superior border of the T7 vertebra on the right side (Figure 2A). This position was considered too deep, so the catheter was subsequently withdrawn by 0.5 cm. However, no repeat x-ray was performed, and it was assumed that the tip had reached a generally accepted safe location. Routine parenteral nutrition infusion was then initiated.

On the second day of life, while on high-frequency oscillatory ventilation, the infant suddenly developed cardiac arrest and poor perfusion, with unobtainable invasive blood pressure and transcutaneous oxygen saturation. Auscultation revealed symmetrical breath sounds in both lungs without rales, and heart sounds were barely audible. Immediate cardiopulmonary resuscitation was performed, including intravenous epinephrine and volume expansion. After 60 s, the heart rate increased to 120 beats per minute, but heart sounds remained muffled.

An immediate POCUS examination was performed. Lung POCUS showed normal lung sliding and B-lines, ruling out pneumothorax; no pleural effusion was seen. Cardiac POCUS revealed a 5.7 mm pericardial effusion at the cardiac apex (Figures 2B,C). Importantly, sonographic surveillance revealed appropriate positioning of the umbilical arterial catheter tip within the descending aorta; conversely, the UVC tip was not clearly visualized. Cranial POCUS showed no intraventricular hemorrhage.

Retrospective review of serial chest radiographs confirmed that the catheter tip had migrated from the T7 level at insertion to the T5 level prior to the acute event. Following the development of pericardial effusion, the UVC likely became wall-apposed, resulting in its failure to visualize on subsequent ultrasonography. This upward migration caused the catheter tip to continuously abut and damage the endothelial lining of the central vein/atrium, leading to gradual fluid extravasation and pericardial effusion. Given the diagnosis of UVC-associated pericardial tamponade, the UVC was promptly removed, and ultrasound-guided pericardiocentesis was performed at the bedside, aspirating 8 mL of hemorrhagic fluid (Figure 2D). After drainage, the infant's heart rate rose to 160 beats per minute, heart sounds became clear, blood pressure improved to 49/27 mmHg, and oxygen saturation stabilized at 93%. Follow-up cardiac ultrasound showed marked reduction of the pericardial effusion (Figure 2E), with no recurrence at 24 and 48 h.

### Case 3

The infant, with a gestational age of 26 + 6 weeks, was transferred to our hospital from an outside institution. The mother did not receive prenatal dexamethasone or magnesium sulfate therapy. The infant had a history of perinatal asphyxia, with Apgar scores of 1, 7, and 8 at 1, 5, and 10 min, respectively. A diagnosis of neonatal respiratory distress syndrome (NRDS) was made, and the infant was treated with surfactant replacement therapy and mechanical ventilation (synchronized intermittent mandatory ventilation [SIMV] mode, with peak inspiratory pressure [PIP] 20 cmH_2_O, positive end-expiratory pressure [PEEP] 6 cmH_2_O, and fraction of inspired oxygen [FiO_2_] 40%).

At 48 h after birth, the Peripheral capillary oxygen saturation (SpO₂) dropped to 70%, leading to a switch to high-frequency oscillatory ventilation (with a mean pressure of 14 cmH₂O, an amplitude of 28 cmH₂O, and FiO₂ 80%), which maintained SpO₂ between 88% and 91%. Laboratory tests revealed a hemoglobin level of 74 g/L and a platelet count of 68 × 10⁹/L. After receiving red blood cell and fresh frozen plasma transfusions, the infant was transferred to our NICU.

**Figure 3:**
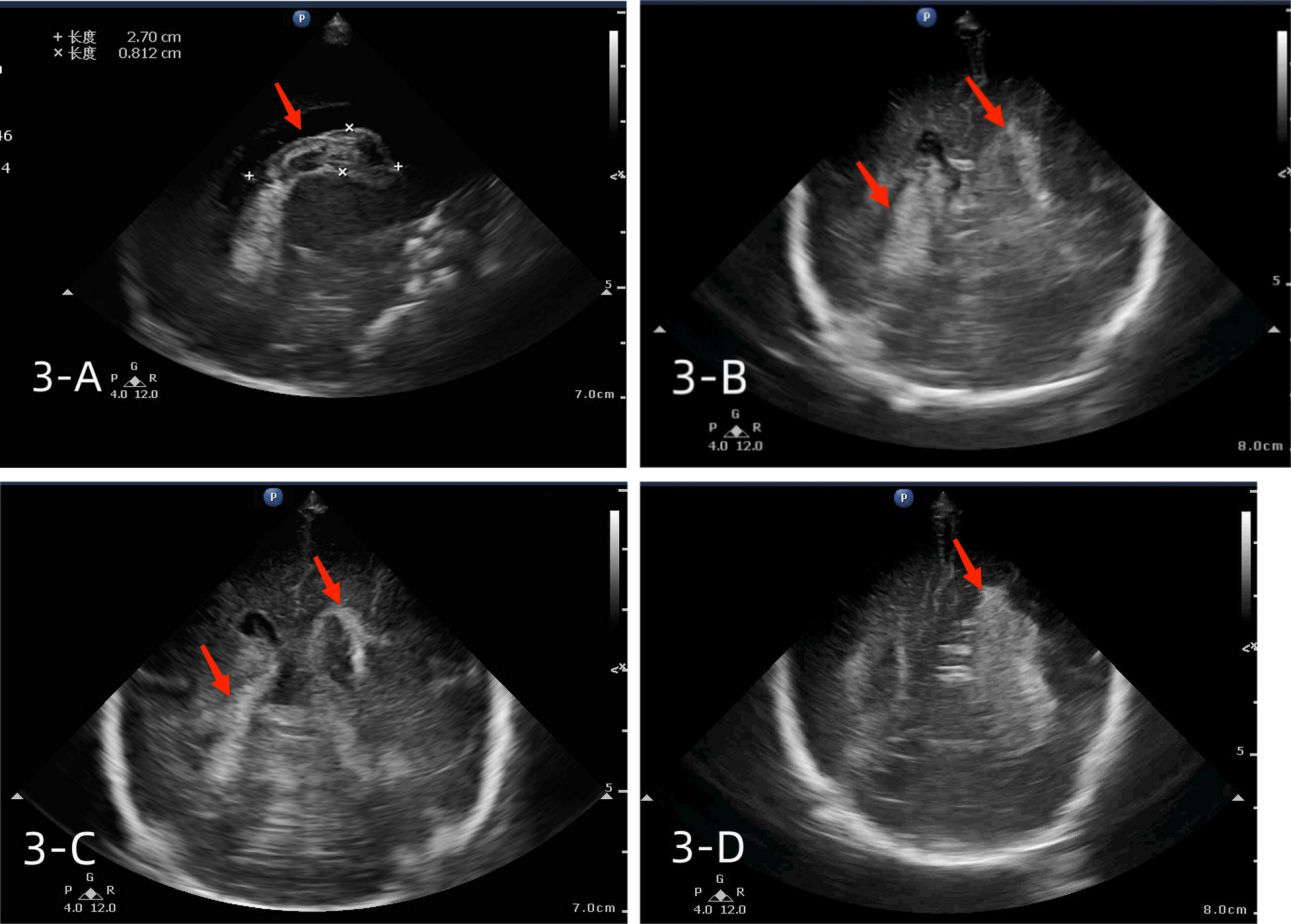
Cranial ultrasound in severe intraventricular hemorrhage. **(A)** Sagittal view showing dilated ventricles filled with echogenic blood (red arrows). **(B–D)** Coronal views demonstrating bilateral ventricular hemorrhage (red arrows) and periventricular echogenicity.

Upon admission, POCUS examination revealed severe intraventricular hemorrhage (Grade IV) (Figures 3A–D). Immediate analgesia and sedation were administered, along with additional red blood cell and fresh frozen plasma transfusions. Following these interventions, the infant's vital signs stabilized, and SpO₂ increased to 93%.

## Discussion

This case series highlights the critical and versatile role of POCUS in diagnosing distinct, life-threatening causes of acute decompensation in neonates (Table 1). In each case, POCUS offered rapid bedside diagnosis that directly accelerated critical clinical management, overcoming limitations of traditional NICU imaging.

**Table 1:** Summary of clinical characteristics, POCUS findings, and outcomes in three neonates with sudden clinical deterioration.

Item	Case 1	Case 2	Case 3
Case No.	1	2	3
Gestational age (weeks)	34	25 + 5	26 + 6
Birth weight (g)	1,660	900	980
Primary diagnosis/Procedure	Postoperative congenital esophageal atresia with tracheoesophageal fistula (type IIIB)	Extremely preterm infant with UVC	Extremely preterm infant with neonatal respiratory distress syndrome (NRDS)
Acute deterioration event	Respiratory distress and hypoxemia 2 h after thoracic drain removal	Sudden cardiac arrest during high-frequency oscillatory ventilation	Acute desaturation and hemodynamic instability on day 2 of life
Key POCUS findings	Absent lung sliding and B-lines on right side, visible “lung point,” stratospheric sign on M-mode	Pericardial effusion with diastolic right ventricular collapse suggestive of tamponade; UVC tip poorly visualized.	Bilateral intraventricular hemorrhage (Grade IV) with ventricular dilation and parenchymal extension
Intervention	Immediate needle decompression and chest tube reinsertion	Ultrasound-guided pericardiocentesis (8 mL hemorrhagic fluid drained)	Supportive care: blood product transfusion, neuroprotective management
Outcome	Effective pulmonary re-expansion achieved, clinical symptoms showed rapid improvement	resolution of effusion on follow-up scan and hemodynamic stabilization	Vital signs stabilized temporarily; transferred for specialist follow-up

Traditional imaging modalities such as chest radiography involve inherent delays, radiation exposure, and the risks associated with transporting unstable neonates (6, 7). POCUS effectively addresses these limitations by offering immediate bedside assessment. Our cases illustrate its pivotal role in specific high-risk NICU scenarios. In Case 1, POCUS performed after a high-risk thoracic procedure (chest tube removal) instantly differentiated tension pneumothorax from other causes of postoperative respiratory collapse, directly guiding emergency needle decompression (4, 5, 8). In Case 2, faced with sudden cardiovascular collapse in an infant with a central venous catheter, cardiac POCUS swiftly identified pericardial tamponade and visualized the malpositioned catheter tip, leading to immediate catheter removal and pericardiocentesis (11, 12), highlighting POCUS's unique ability to detect dynamic catheter migration that static x-ray could not capture. To avoid complications arising from such risks, our hospital is gradually transitioning from standard blind catheter placement confirmed by CXR to real-time POCUS-guided UVC insertion. This technique allows direct visualization of the catheter tip position at the cavoatrial junction, significantly reducing the risk of such catastrophic complications. For Case 3, cranial POCUS upon admission of an extremely preterm infant with hemodynamic instability promptly revealed a severe IVH, redirecting management towards neuroprotective strategies and explaining the clinical deterioration (13, 14). This rapid, precise bedside diagnosis directly guides life-saving interventions and avoids delays and risks from transporting unstable infants.

**Figure 4:**
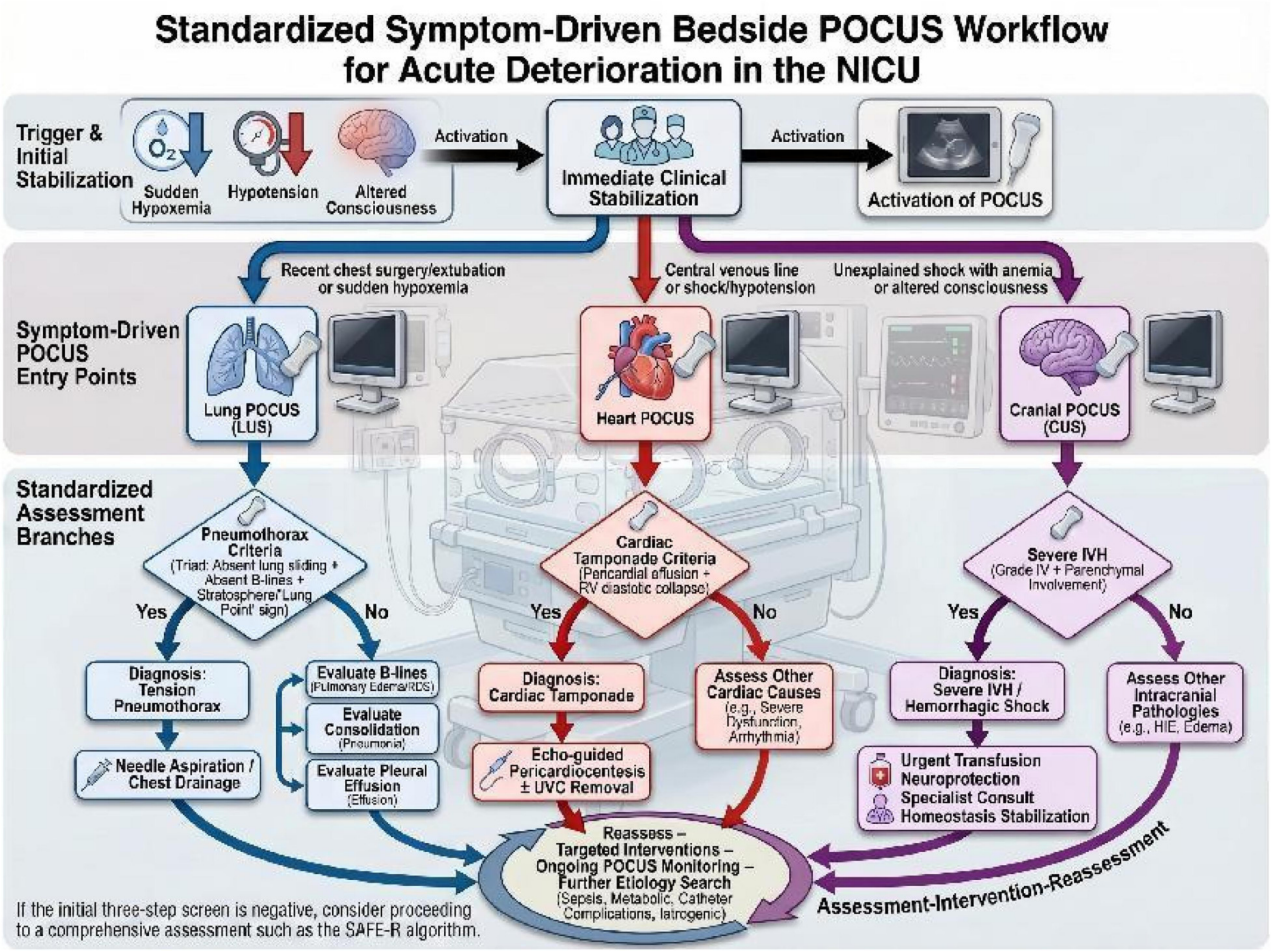
A structured POCUS screening pathway for the crashing neonate in the NICU. The algorithm outlines a systematic, bedside approach involving rapid sequential examination of the lungs, heart, and brain to expedite the diagnosis or exclusion of critical conditions: tension pneumothorax, pericardial tamponade, and severe IVH. LUS, lung ultrasound; CUS, cranial ultrasound.

Managing these three emergencies led to the development of a clear, structured POCUS screening protocol for critically ill neonates (Figure 4). We propose a sequential “heart-lungs-brain” approach, focusing on the key anatomic sites of life-threatening pathology in neonates (4, 15). Compared to the existing SAFE-R algorithm (9), our pathway offers a streamlined assessment applicable to all neonates with acute cardiopulmonary decompensation, while its low-pressure, minimally invasive design offers particular advantages for ELBW infants given their unique fragility and vulnerability to iatrogenic injury.The feasibility of routine abdominal screening in crashing ELBW infants warrants specific caution. Applying probe pressure to a fragile abdominal wall to assess the aorta or free fluid prolongs the procedure and may increase the risk of germinal matrix hemorrhage-IVH, as procedural stress and hemodynamic instability are well-established risk factors for IVH in preterm infants (16, 17). Furthermore, acute abdominal catastrophes rarely present as sudden cardiopulmonary collapse without antecedent clinical signs (18, 19). Thus, our algorithm intentionally reserves abdominal evaluation as a second-tier step if the initial “heart-lungs-brain” screen is negative, optimizing the risk-benefit ratio during high-stakes resuscitations. As evidenced by our cases, the order can be tailored: lung ultrasound was paramount in primary respiratory failure (Case 1), while cardiac ultrasound was the immediate priority in cardiovascular collapse (Case 2). Our early experience implementing this pathway highlights two practical prerequisites for success in a resuscitation scenario: clear pre-assigned roles within the team for clinical stabilization and POCUS acquisition, and immediate access to a dedicated ultrasound machine at the bedside to minimize workflow disruption. This proposed pathway provides a pragmatic, stepwise framework intended to reduce diagnostic uncertainty and improve time-to-intervention during high-stakes neonatal emergencies.

The effective use of POCUS as demonstrated here requires structured training and competency assessment. Evidence indicates neonatologists can acquire these essential skills through focused programs (20, 21). In our institution, a progressive curriculum beginning with lung ultrasound has enhanced proficiency, an approach now reinforced by the recent expert consensus on standardizing lung ultrasound training for neonatologists (22). This consensus underscores the foundational role of structured lung ultrasound education within neonatal POCUS competency frameworks, further validating its prioritization as the first step in our proposed ‘heart-lungs-brain’ pathway. However, widespread implementation faces recognized barriers, including equipment costs, the need for dedicated training time, and the operator-dependent nature of the technique. Developing standardized, accessible training modules and certification pathways, as suggested by international guidelines, is crucial for safe and responsible adoption (23, 24).

This case series corroborates and extends the existing literature on POCUS in neonatal emergencies. Previous studies have established POCUS's diagnostic accuracy for specific conditions like pneumothorax (4), a critical event with well-documented risk factors and severe potential outcomes in neonates (25). Our work extends this by systematically demonstrating its rapid discriminative utility across three lethal complications: pneumothorax, tamponade, and severe IVH, within a unified emergency assessment narrative. Additionally, we provide a practical example of dynamic POCUS monitoring for post-extubation pneumothorax following major thoracic surgery, emphasizing its role in surgical patient follow-up imaging. Finally, we highlight its immediate diagnostic value in a rare but critical iatrogenic complication—UVC-associated tamponade in an ELBW infant—where rapid diagnosis is vital for survival and the prevention of sudden cardiac death (26). This integrated presentation and the proposed structured screening approach aim to elevate POCUS from an optional skill to an essential competency in the NICU.

This study has several limitations. Its design as a single-center, retrospective case series with a small sample size introduces potential selection bias; prospective, larger-scale studies are needed to validate the conclusions. Second, POCUS diagnosis is operator-dependent. Although trained physicians performed all studies here, inter-observer variability remains a consideration. Future studies could employ blinded dual assessments to enhance reliability. Furthermore, our focus was on short-term diagnosis and intervention; we lack data on the long-term neurodevelopmental outcomes of these infants. Future research should prioritize establishing standardized POCUS training and certification programs for neonatal emergencies. In addition, multi-center studies are needed to compare the cost-effectiveness and diagnostic accuracy of POCUS with conventional imaging pathways. Finally, exploring the potential of artificial intelligence-assisted POCUS image recognition represents a promising direction. Furthermore, future multi-center studies should be conducted to validate the generalizability and clinical impact of our proposed algorithm.

## Conclusion

POCUS is a valuable adjunct in managing critically ill neonates who experience sudden clinical deterioration. It enables rapid etiological diagnosis and directly guides time-sensitive interventions. As illustrated in our cases, bedside POCUS successfully differentiated tension pneumothorax, pericardial tamponade, and severe intracranial hemorrhage, directly altering the clinical management trajectory and avoiding the risks associated with conventional imaging delays. We advocate for the adoption of structured, standardized POCUS screening protocols during neonatal resuscitation. Furthermore, we support ongoing efforts to establish unified training and certification pathways, ensuring that POCUS serves as an essential, safe, and integrated component of modern neonatal critical care.

## Data Availability

The original contributions presented in the study are included in the article/Supplementary Material, further inquiries can be directed to the corresponding authors.
